# Rigor Me This: What Are the Basic Criteria for a Rigorous, Transparent, and Reproducible Scientific Study?

**DOI:** 10.3389/fcvm.2022.913612

**Published:** 2022-07-01

**Authors:** Brian E. Sansbury, Matthew A. Nystoriak, Shizuka Uchida, Marcin Wysoczynski, Joseph B. Moore

**Affiliations:** ^1^Diabetes and Obesity Center, University of Louisville School of Medicine, Louisville, KY, United States; ^2^Center for RNA Medicine, Department of Clinical Medicine, Aalborg University, Copenhagen, Denmark

**Keywords:** preclinical *in vivo* studies, preclinical *in vitro* studies, rigor and reproducibility, rigor and bias in research, data reporting standards

## Abstract

Scientific advancement is predicated upon the ability of a novel discovery to be independently reproduced and substantiated by others. Despite this inherent necessity, the research community is awash in published studies that cannot be replicated resulting in widespread confusion within the field and waning trust from the general public. In many cases, irreproducibility is the unavoidable consequence of a study that is conducted without the appropriate degree of rigor, typified by fundamental flaws in approach, design, execution, analysis, interpretation, and reporting. Combatting the irreproducibility pandemic in preclinical research is of urgent concern and is the primary responsibility of individual investigators, however there are important roles to be played by institutions, journals, government entities, and funding agencies as well. Herein, we provide an updated review of established rigor criteria pertaining to both *in vitro* and *in vivo* studies compiled from multiple sources across the research enterprise and present a practical checklist as a straightforward reference guide. It is our hope that this review may serve as an approachable resource for early career and experienced investigators alike, as they strive to improve all aspects of their scientific endeavors.

## Introduction

Widespread irreproducibility of scientific studies is perhaps the most vexing issue impacting modern biomedical research today. It is a veritable problem with manifold consequences on the scientific and medical community, as well as the general public. The publication and dissemination of methodologically flawed or suboptimal research significantly limits scientific advancement, impedes the clinical translation of pre-clinical findings, erodes public confidence in the results of scientific/medical studies and the public health policies they may inform. Beyond these obvious consequences, non-rigorous research may also contribute to misguided apportionment of monetary support and wasteful spending of finite research funds for projects whose rationale is derived from false, canonized scientific facts. While this issue of irreproducibility in preclinical research and its harms are well-recognized in the academic community and biopharmaceutical industry at large (1–5), disconcertingly, methodological deficiencies have remained prevalent in preclinical research (6–9). Though the causes of irreproducibility are most certainly multifactorial and have been discussed at great lengths in recent years (4, 7, 10–16), deficiencies in the practice of methodological rigor are arguably the leading contributor. Recognizing this, funding agencies and editors from leading journals have worked assiduously to establish and implement new reporting standards to boost scientific rigor of published works (1, 15, 17–26). With what appears to be an ever-expanding list of rigor standards and reporting guidelines to put into practice, the question for some, especially those early careered scientists, is what are the basic criteria one must prioritize and incorporate in their preclinical study to ensure that it is scientifically sound, transparent, and reproducible? In consideration of this important question, the current editorial will draw from current principles and guidelines for rigor and reproducibility established by leading journals, scientific leaders, and funding agencies to forward a practical and abbreviated guide that details basic criteria necessary to assure scientific rigor in preclinical studies.

The guide described herein is closely modeled after editorial checklists established by *Stroke* (12) and *Circulation Research* (18) to document and enhance the methodological rigor of preclinical work upon publication; additionally, it incorporates rigor criteria promulgated by the NIH (15) and ARRIVE (Animal Research: Reporting of *in vivo* Experiments) (19–28). Here, rigor guideline items are stratified according to reporting criteria for molecular and cellular studies *in vitro* and experimental interventions involving animal models *in vivo*—specifically for reasons that both settings present with a number of distinct challenges and, thus, have unique methodological and reporting criteria. The key methodological and reporting characteristics (denoted by roman numerals in subsequent sections) and their procedural descriptions should not be viewed as a mere list of scientific rigor items to simply state as “included” or “performed” in a completed study, but are instead a set of fundamental scientific principles and reporting standards that should be routinely utilized in both the *a priori* design and technical execution of pre-clinical studies—all elements of which should be clearly and accurately reported in the text of published works.

## Rigor Fundamentals

### Reporting Criteria for Molecular and/or Cellular Studies (*in vitro*)

Both the interrogation of biological phenomena and the delineation of molecular mechanisms contributing to cellular and organismal physiology often requires the performance of molecular and cellular experiments *in vitro*. Naturally, data robustness and the validity of conclusions drawn from such experiments (or all experiments for that matter) require that they be well-designed, carefully controlled, appropriately powered, and adequately described. Nevertheless, while these principles are arguably the most fundamental in experimental practice, methodological weaknesses, and substandard reporting remain a source of experimental irreproducibility in this research domain (29). Fortunately, efforts led by the editors of some of the world's premiere cardiovascular journals, including *Stroke* and *Circulation Research* (12, 18), and a number of scientific agencies (15, 19–28), have facilitated the establishment of reporting standards or rather reporting checklists meant to remedy ongoing reporting deficiencies. Concerning molecular and/or cellular studies performed *in vitro*, such rigor guidelines highlight a set of methodological and reporting characteristics that fall within four core topic areas. These include Study Conduct, Data Reporting, Statistical Procedures, and Data Availability (outlined in Table 1)—each of which are briefly discussed below.

**Table 1:** Molecular and cellular studies (*in vitro*).

**Methodological and reporting characteristics**	**Key reporting items**
I. Study conduct	□ Characteristics of cell lines used (e.g., source, authentication, and passage number)
	□ Characteristics of antibodies employed (e.g., source, host, immunoglobulin fragment, titer, catalog number, and how the antibody was validated)
	□ Description of replicates (e.g., sufficient information concerning sample collection is provided to distinguish between biological and technical replicates)
	□ Data processing, data conversions, and experimental metadata are described
II. Data reporting	□ Description of methodology (of sufficient detail to allow replication)
	□ Immunoblots (experimental and controls groups are included in the same blot, molecular weight standards are included, etc.)
	□ Immunofluorescence (images are similarly processed in experimental and control groups, scale bars are provided in all presented micrographs, etc.)
	□ All experimental controls are included and described
	□ Presentation of tabulated data (use of graphs which afford visualization of data distribution characteristics)
	□ Units of measure are specified
III. Statistical procedures	□ Description of statistical procedures
	□ The use of formal tests for normality
	□ Multiple hypotheses testing
IV. Data availability	□ Data access statements are provided (i.e., data was made available in a public data repository)

*Checklists for the reporting of molecular and cellular studies, adapted from journal rigor guidelines originally designed by Stroke in 2011*.

### Study Conduct

*What biological materials were used, how were experimental replicates defined, and how were resultant data collected/processed?* Manuscripts should include explicit details concerning all biological materials (i.e., cell lines) and/or reagents (e.g., antibodies, pharmacologic agents, small molecule inhibitors, etc.) utilized in experiments. These include characteristics of cell lines, such as their source (vender or laboratory in which they were derived), authentication, and passage number (especially at the time they were used in experiments). Characteristics of all antibodies employed in experiments (i.e., primary antibodies, secondary antibodies, isotype control antibodies, etc.) should also be reported in the text. Such information should include their source, host, and immunoglobin titer; however, if obtained from a commercial entity, one may only need provide a manufacturer catalog number (so long as the aforementioned information has been made available from the source company). Beyond providing the basic characteristics of experimental accouterments, it is critical that authors provide a complete description of experimental replicates. More specifically, sufficient information regarding sample collection should be provided so that readers may distinguish between biological and technical replicates. Finally, data processing, data conversions (i.e., changing computer data from one format to another), and experimental metadata (e.g., instrument settings and configuration, etc.) should also be described.

### Data Reporting

*How were experiments performed and how should digital images and tabulated data be displayed?* In all submitted works, methods should be of sufficient detail to allow full replication of the study—even for those procedures that may be considered pedestrian or routine (i.e., H&E staining, immunoblotting, etc.). In general, it is not unreasonable to refer to previously published procedures or manufacturer protocols (especially in the case where a kit has been used); however, any deviations should be detailed in the text. Beyond an accurate and thorough description of methods, there exists a number of unique considerations for the reporting of various types of data. For immunoblots, both experimental and controls groups must be included in the same blot. What is more, all presented immunoblots should contain markers denoting the location of molecular weight standards that were electrophoretically resolved with experimental protein lysates. One must also routinely consider the inclusion of controls to validate antibodies used in Westerns. These may include blocking or immunizing peptides, cell/tissue plus/minus expression controls, etc. While not all journals adhere to the same set of standards, it is also prudent for authors to always have available all unedited immunoblots generated throughout the course of their study as they may be requested as supplemental materials by a journal or manuscript reviewers. With regard to immunofluorescence images, experimental and control groups must be similarly processed. Immunostaining images should also be accompanied by a detailed set of controls, which include those designed to validate antibody specificity (i.e., isotype control antibodies, tissue plus/minus controls, etc.) and distinguish genuine target staining from background (i.e., secondary antibody only controls, etc.). Lastly, all presented micrographs should contain a decipherable scale reporting both scale dimensions and units of measure.

Whilst there exist numerous types of graphs to choose from when presenting tabulated data, authors should consider the use of graphs that afford readers the ability to visualize data distribution characteristics and variability in experimental replicates. Dot plots, violin plots, and box plots are increasingly preferred by many journals for reasons that readers can more ably interpret the distribution, range, shape, and skewness of numerical data. Traditional bar graphs, especially those reporting only standard error of the mean (SEM), provide little insight concerning the variability and shape/distribution of a set of data (30). Finally, authors should be careful to ensure that units of measure are specified for all tabulated and graphically presented numerical data.

### Statistical Procedures

*How were data analyzed and what programs were used?* A separate section should be dedicated to the description of statistical procedures utilized for each data set in the study. This may include a description of statistical software/programs utilized in analyses, tests for statistical significance employed, *post hoc* multiple comparisons tests used in the acquisition of corrected *p*-values, etc. In addition, authors should ensure that the central tendency (i.e., mean, median, mode) and dispersion (e.g., range, variance, standard deviation, standard area) of all data sets are examined and reported in the text.

All too often are data distribution characteristics overlooked in published studies; in many instances, parametric statistical procedures are used on data sets that have not been confirmed to be normally distributed or contain too few replicates to reliably assess data distribution characteristics. Accordingly, all data should be subjected to formal tests for normality (specify those tests used in the manuscript). Data not following a normal distribution should be analyzed via non-parametric procedures or mathematically transformed prior to using parametric tests for statistical significance. Lastly, for those studies that are not exploratory, corrections for multiple hypotheses testing [i.e., adjusting *p*-values to account for multiple testing (31)] and multiple comparisons should be performed and mentioned in the text.

### Data Availability

*Have the study's generated datasets been made publicly available, and if so, where might they be accessed?* Although not universally mandated by scientific journals as part of the manuscript submission process, it is good practice to ensure that all “omics” data (transcriptomics, epigenomics, lipidomics, proteomics, metabolomics, etc.) generated in the study be made available in a public data repository that is supported by a research community or research institution. Data access statements should be explicitly provided in the manuscript's text and include the name/location of the data repository to which data was submitted and corresponding dataset accession numbers. Simply saying that “data are available from the corresponding author upon reasonable request” provides no real assurance that the study's data will be accessible to the research community.

### Reporting Criteria for Experimental Interventions in Animals (*in vivo*)

Animal models have long been exploited in basic science and preclinical research and have proven themselves as indispensable tools in understanding complex cellular and physiologic processes. Without question, animal models have afforded invaluable information in the quest of knowledge concerning the pathophysiology of human disease and the discovery of therapeutic agents. While they have proven to be a great asset to the scientific community, animal-based studies remain arguably one of the most impacted by methodological and reporting deficiencies (11, 32). In fact, it was previously estimated that between 51 and 89% of animal studies cannot be reproduced (33). Widespread awareness of such issues inevitably led to numerous calls for action. In response, the National Centre for the Replacement, Refinement and Reduction of Animals in Research (NC3Rs) developed the ARRIVE guidelines in 2010 (19–28)—a set of reporting criteria designed to enhance the standard of reporting on research using animals. These guidelines, which have experienced recent revisions and updates (26), are endorsed by the American Heart Association and its operated journals (e.g., *Stroke* and *Circulation Research*) and are incorporated into their mandated preclinical checklists. These checklists, harboring reporting criteria for experimental interventions in animals, detail a series of methodological and reporting characteristics that fall within eight core topic areas. These include Study Design Elements, Inclusion/Exclusion Criteria, Randomization, Blinding, Sample Size Determination, Data Reporting, Statistical Procedures, and Ethics & Funding Statements (outlined in Table 2)—each of which are briefly discussed below.

**Table 2:** Experimental interventions in animals (*in vivo*).

**Methodological and reporting characteristics**	**Key reporting items**
I. Study design elements	□ Experimental groups are defined
	□ Study timeline is provided
	□ Primary and secondary endpoints are specified
	□ Sex was considered as a biological variable
II. Inclusion/exclusion criteria	□ Inclusion and exclusion criteria for animal enrollment are defined
	□ Criteria were set *a priori* (prior to carrying out the study)
III. Randomization	□ Animals were randomly assigned to experimental groups
	□ Type and methods of randomization are described
IV. Blinding	□ Blinding procedures were used in the study
	□ Description of blinding procedures is provided (how they were performed and in what experiments they were used)
V. Sample size determination	□ A priori power calculations were used in the determination of group sizes
	□ Power calculations are described
VI. Data reporting	□ Baseline characteristics of animals (species, age, sex, strain, chow and bedding, and source)
	□ The number of animals in each group that were randomized, evaluated, and excluded from the study is specified
	□ Baseline data on measured endpoints are reported for all groups
	□ Adverse events that occurred during the course of the study are reported
	□ Tabulated data is presented (use of graphs which afford visualization of data distribution characteristics)
	□ Numeric data on outcomes are provided in the text or in a tabular format (in addition to figures)
	□ Methodology is appropriately described (of sufficient detail to allow replication)
	□ Animal procedures/handling are thoroughly described (e.g., formulation/dosage of therapeutic agents, site and route of administration, temperature control during procedures, postprocedural monitoring, etc.)
VII. Statistical procedures	□ Statistical procedures are described
	□ Data Distribution was assessed
	□ Multiple hypotheses testing
	□ In negative studies, the probability of type II error is reported
VIII. Ethics & funding statements	□ Statements on approval by ethics boards and ethical conduct of studies are provided
	□ Statements on funding and conflicts of interests are provided
	□ Author contributions are described

*Checklists for the reporting of experimental interventions in animals, adapted from journal rigor guidelines originally designed by Stroke in 2011*.

### Study Design Elements

*Is the study design clearly and accurately articulated in the document?* Authors should ensure that study design elements are appropriately described and without ambiguity. All experimental groups and the number of animals in each experimental arm should be clearly stated in the text. One must also ensure an adequate description of the control group(s) and their relationship to that of the treatments (i.e., whether or not they were matched). Authors should confirm that the overall study timeline is provided and, further, that primary and secondary endpoints are specified. Importantly, authors must also explicitly indicate whether sex was considered as a biological variable in the study. If not, the rationale for the preferential use of one sex over the other should be provided.

### Inclusion/Exclusion Criteria

*What were the criteria for animal inclusion and exclusion from the study and were they established a priori?* Animal inclusion and exclusion criteria for animal enrollment into the study must be defined in the manuscript. More specifically, authors must specify those requisite baseline phenotypic characteristics or biological parameters necessary for an animal to be included in the study. In a similar manner, authors should indicate whether any animals were excluded from any analyses. If so, authors should clearly define and discuss the criteria according to which such animal exclusions were made—especially as they relate to interrogated primary and secondary endpoints. Lastly, one should also make clear in the manuscript whether these criteria were established *a priori* (i.e., before commencing the study).

### Randomization

*Were animals randomly assigned to experimental groups?* In addition to ensuring that each experimental arm has an equal probability of receiving a specific treatment, the use of appropriate randomization procedures when assigning animals to experimental groups effectively aids in the prevention of selection bias. Thus, it comprises a critical rigor element of a well-designed animal-based study, which warrants a clear description of methods and procedures employed. In submitted works, authors should indicate whether animals were randomly assigned to experimental groups and, if so, provide details concerning the type and methods randomization that were used (e.g., random number generator, etc.). If random assignment was not used, an adequate explanation/justification should be provided in the text. Further, authors should state whether allocation concealment was used (i.e., hiding the treatment to be allocated to each animal from the individuals assigning the animals to experimental groups) and the methods or means by which this was performed.

### Blinding

*Were data collected and/or analyzed in a blinded fashion?* Perhaps one of the most effective means to combat bias is through the routine use of blinding or masking procedures. Despite its importance, one may not be surprised to find that blinding is not consistently mentioned or described in many published pre-clinical studies. Authors should ensure that blinding procedures, such as masking of group/treatment assignment from the experimenter, is used and described in the text of submitted. In those rare instances that the experimenter was not blinded or where the experimenter was able to decipher group/treatment assignment (e.g., a compound/solution to be administered has a discernible color, odor, or turbidity), written explanation and/or clarification should be provided. Moreover, additional blinding safeguards, which may include the masking of group assignment, genotype, and/or treatment during outcome assessment must also be discussed in the manuscript's text. Again, for any reason individuals were not blinded during outcome assessment and/or data analyses, the rationale for not doing so should be disclosed.

### Sample Size Determination

*How were group sizes determined in the study?* Power and sample size calculations are necessary to determine the minimal number of subjects or animals required to answer the research question. In all preclinical studies involving animals, formal sample size and power calculations should be conducted before commencing the study. These power calculations should be explicitly described in the text of all submitted works and based on a priori determined outcomes and treatment effects. Should power calculations have not been performed, authors should provide a rationale for not doing so, as well as disclose alternative means used in the determination of group sizes in their study. Merely stating alone that “the choice of group size is based on prior experience” or “the numbers used are consistent with previous studies in the field” provides little assurance that the study was sufficiently powered to detect an effect, if a true effect existed to detect.

### Data Reporting

*How were experiments performed, what are the characteristics of the animals used, and how should resultant data be reported?* It is ideal to include a section dedicated to the reporting of animal baseline characteristics. These include species, strain, age or age range, sex, and source (i.e., vender or laboratory) of animals used in experiments. Further, it is also prudent to describe the animal housing/environment, which may include information concerning type and access to food and water (i.e., *ad libitum*, timed, etc.), bedding, light cycle durations, etc. Research manuscripts shall also report the number of animals in all groups that were randomized, tested, and excluded from analyses (including those that died). Beyond this, authors should disclose details regarding any adverse events and/or deaths that occurred during the course of the study for all experimental groups.

It is ideal to report numerical data on outcomes in multiple ways—such as graphically, in the text, and/or in a tabular format (tables); reason being that tables are best suited for referencing specific information, while graphs are ideal for the visual interpretation of trends and making group comparisons. As previously discussed, authors should consistently use graphs that enable the visualization of data distribution characteristics and experimental replicate variability, such as dot plots or equivalent. Finally, all methods should be of sufficient detail to allow complete replication of the study, including all aspects of animal handling and treatment (e.g., formulation and dosage of therapeutic agents, site and route of administration, temperature control during procedures, post-procedural monitoring, etc.).

### Statistical Procedures

*How were data analyzed and how should they be reported?* As discussed earlier, a separate section should be dedicated to the description of all statistical methods and procedures used in the analyses. Further, for each statistical test performed, the effect size with its standard error and *p*-value should be reported in the text. Whilst not typically required by many scientific journals, it is also prudent for authors to provide confidence intervals for vital comparisons. Authors must also ensure that the central tendency and dispersion of all data sets are examined and reported in the text (especially for small data sets). All data should be subjected to formal tests for normality and those tests used reported in the manuscript. If studies are not hypothesis-generating or exploratory in design, corrections for multiple hypotheses testing and multiple comparisons should be performed and detailed in the text. Finally, for studies that are negative or do not have statistically significant findings, the probability of a type II error should be reported.

### Ethics and Funding Statements

*Did the study receive pertinent approvals and who contributed to the work?* Authors must not overlook the inclusion of statements indicating that the study conforms to appropriate regulations and guidelines relating to the use of animals. Said statements should include the name of the institution in which the research was approved and the ethics agencies that reviewed it. Further, one should include statements detailing all sources of financial support for the study, as well as any substantive author conflicts of interests (intellectual or monetary). Finally, authors should provide statements regarding author contributions. While not universally required information, a number of scientific journals encourage transparency through disclosing author-specific contributions (e.g., conception or design of the study, data collection, procurement of study materials, data analysis and interpretation, drafting the article, etc.). These statements are important as they not only allow readers the ability to discern the role(s) of each author in its design, execution, and interpretation, but also ensures that those included have made substantive contributions meriting authorship.

## Concluding Remarks

Whilst the root causes of data irreproducibility are many, the major contributors, such as deficiencies in the practice of methodological rigor and incomplete or inaccurate reporting, are in our direct control. The routine and judicious use of standardized rigor/reporting guidelines are a logical remedy to these issues. In fact, the prescribed use of journal reporting guidelines & checklists and the introduction of technical reviewers (34) (responsible for ensuring author compliance) has proven to be efficacious (9). While the benefits of this rigor framework have been realized in the scientific community, these alone will not safeguard against scientific irreproducibility stemming from intentional dishonesty and/or fraud—just as data can be intentionally falsified, so too can rigor criteria. Though perhaps not “the panacea” for all cases of scientific irreproducibility, following the rigor guidelines presented here and ensuring their clear and accurate description in submitted works will make it easier for the scientific community to either reproduce research findings or refute them.

## Author Contributions

BS, MN, SU, MW, and JM: conceptualization and writing—review and editing. BS, MN, and JM: writing—original draft. JM: funding acquisition. All authors contributed to the article and approved the submitted version.

## Funding

This work was supported by the National Institutes of Health [Grant No: R01 HL141081]. This work was also supported in part by a grant from the Jewish Heritage Fund for Excellence Research Enhancement Grant Program at the University of Louisville, School of Medicine.

## Conflict of Interest

JM currently serves as the senior technical editor for *Circulation Research* journal. The remaining authors declare that the research was conducted in the absence of any commercial or financial relationships that could be construed as a potential conflict of interest.

## Publisher's Note

All claims expressed in this article are solely those of the authors and do not necessarily represent those of their affiliated organizations, or those of the publisher, the editors and the reviewers. Any product that may be evaluated in this article, or claim that may be made by its manufacturer, is not guaranteed or endorsed by the publisher.
